# Association Between Triglyceride‐Glucose Index and in‐Hospital Intensive Care Unit Readmission in Ischemic Stroke Patients: A Retrospective Analysis Based on the MIMIC‐IV Database

**DOI:** 10.1002/kjm2.70250

**Published:** 2026-06-16

**Authors:** Di Zhang, Ying‐Lun Chen, Jian Zhang, Xiang‐Jun Hu

**Affiliations:** ^1^ Department of Rehabilitation Medicine, Shanghai Geriatric Medical Center, Zhongshan Hospital Minhang Branch Fudan University Minhang Hospital Shanghai China; ^2^ Department of Rehabilitation Medicine Zhongshan Hospital, Fudan University Shanghai China

**Keywords:** intensive care unit, ischemic stroke, MIMIC‐IV database, readmission, triglyceride‐glucose index

## Abstract

This retrospective cohort study utilized the Medical Information Mart for Intensive Care database to investigate the association between the triglyceride–glucose (TyG) index and in‐hospital intensive care unit (ICU) readmission in patients with ischemic stroke (IS). Among 2929 patients, 24.0% experienced ICU readmission. Logistic regression analysis demonstrated that the continuous TyG index was associated with a 17% higher risk of ICU readmission (odds ratio [OR], 1.17; 95% confidence interval [CI], 1.01–1.36; *p* = 0.04). Compared with the lowest quartile (Q1), the higher TyG index quartiles showed a progressively increased risk of ICU readmission (Q2: OR, 1.49; 95% CI, 1.13–1.96; Q3: OR, 1.46; 95% CI, 1.10–1.95; Q4: OR, 1.53; 95% CI, 1.14–2.05; *P* for trend = 0.02). Restricted cubic spline analysis revealed a significant nonlinear relationship (*P* for nonlinearity = 0.027), with an inflection point at a TyG index value of 9.82 (*P* for log‐likelihood ratio = 0.007). Incorporating the TyG index into conventional models significantly improved the predictive performance for ICU readmission, as evidenced by increases in the area under the curve, net reclassification improvement and integrated discrimination improvement. In conclusion, an elevated TyG index was associated with an increased risk of in‐hospital ICU readmission in patients with IS and exhibited a nonlinear dose–response relationship. The TyG index enhanced the predictive accuracy of existing scoring tools, suggesting its potential clinical value for risk stratification and early intervention.

**Abbreviations**
Alb: albumin
ALT: alanine aminotransferase
APS III: Acute Physiology Score III
APTT: activated partial thromboplastin time
AST: aspartate aminotransferase
AUC: area under the receiver operating characteristic curve
BUN: blood urea nitrogen
CCI: Charlson Comorbidity Index
CI: confidence interval
DBP: diastolic blood pressure
DCA: decision curve analysis
GCS: Glasgow Coma Scale
Glu: glucose
Hb: hemoglobin
HDL‐C: high‐density lipoprotein cholesterol
HR: heart rate
ICD: International Classification of Diseases
ICU: intensive care unit
IDI: integrated discrimination improvement
INR: international normalized ratio
IQR: interquartile range
IR: insulin resistance
IS: ischemic stroke
LASSO: Least Absolute Shrinkage and Selection Operator
MIMIC‐IV: Medical Information Mart for Intensive Care
NRI: net reclassification improvement
OASIS: Oxford Acute Severity of Illness score
OR: odds ratio
PLT: platelet count
RBC: red blood cell count
RCS: restricted cubic spline
SBP: systolic blood pressure
Scr: serum creatinine
SD: standard deviation
SOFA: Sequential Organ Failure Assessment
TC: total cholesterol
TG: triglycerides
TyG: triglyceride–glucose index
VIF: variance inflation factor
WBC: white blood cell count

## 1 Introduction

Ischemic stroke (IS) is a major global health challenge and is associated with substantial mortality and long‐term disability [1]. In acute IS care, 15.0%–20.0% of patients require intensive care unit (ICU) admission during the initial hospitalization due to severe neurological deficits or systemic complications [2]. Some patients subsequently deteriorate after transfer to the ward and require ICU readmission, which is associated with worse outcomes and an increased healthcare burden [3, 4, 5]. Therefore, early identification of patients at risk for ICU readmission is critical [1, 4].

The triglyceride–glucose (TyG) index, calculated from fasting blood glucose (Glu) and triglyceride levels, is a validated surrogate marker of insulin resistance (IR) and has been shown to predict the risk of incident IS in the general population [6, 7]. Substantial evidence has linked an elevated TyG index to adverse outcomes in patients with IS, including stroke recurrence, functional impairment, and mortality, particularly among critically ill individuals [8, 9, 10]. In severe IS, recurrent in‐hospital deterioration indicates critical clinical status and unfavorable neurological outcomes [11]. ICU readmission following ward transfer independently predicts prolonged hospitalization, increased mortality, and higher healthcare costs, thereby highlighting the need for early risk identification [11, 12]. Previous studies have primarily focused on the TyG index as a predictor of post‐discharge recurrence or readmission. However, the association between the TyG index and in‐hospital ICU readmission in critically ill patients remains unclear.

Therefore, this study aimed to investigate whether the TyG index is independently associated with ICU readmission during hospitalization in patients with IS using the Medical Information Mart for Intensive Care (MIMIC‐IV) database and to evaluate its incremental predictive value beyond established severity scores.

## 2 Material and Methods

### 2.1 Data Source

This retrospective cohort study utilized data from the MIMIC‐IV database (version 2.2), which contains de‐identified records of critically ill patients admitted to Beth Israel Deaconess Medical Center between 2008 and 2019 [13]. Ethical approval and informed consent were waived because the database contains anonymized data. The author was granted access to the database after completing the Collaborative Institutional Training Initiative examination (certification number: 66647616).

### 2.2 Study Population

**FIGURE 1:**
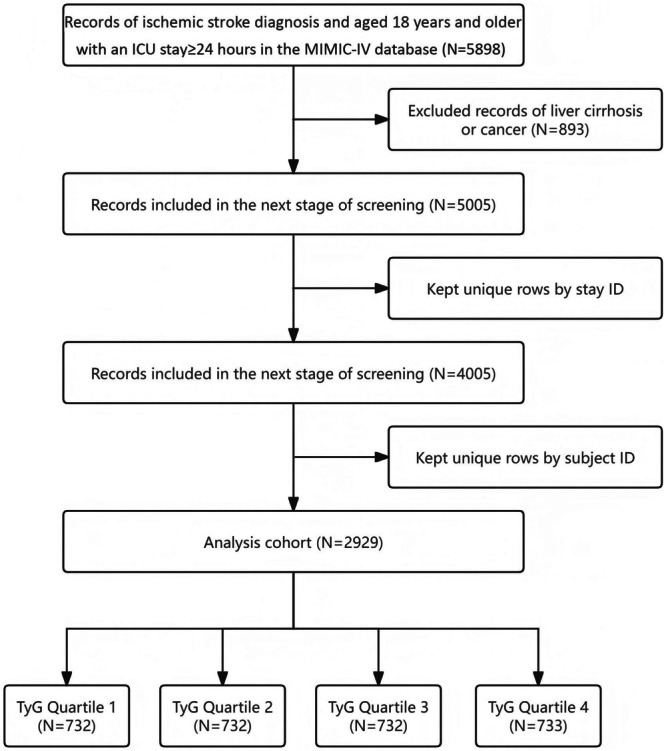
Flowchart for patients.

The study included patients diagnosed with IS, as defined by the International Classification of Diseases, Ninth and Tenth Revisions (ICD‐9 and ICD‐10). We restricted the analysis to the first ICU admission for patients with multiple hospital admissions but retained all ICU stays within a single hospitalization. The exclusion criteria were as follows: (1) age < 18 years, (2) length of ICU stay < 24 h, and (3) a diagnosis of liver cirrhosis or cancer. After excluding 893 records, 5005 records remained. After deduplication, 2929 unique patients were included in the final analysis. These patients were subsequently categorized into TyG index quartiles: Q1 (7.1–8.6), Q2 (8.6–9.0), Q3 (9.0–9.4), and Q4 (9.4–13.1) (Figure 1).

### 2.3 Data Collection

Demographic characteristics, severity‐of‐illness scores, laboratory data, vital signs, comorbidities, treatments, and clinical outcomes were extracted from the database. Demographic variables included age, sex, height, weight, marital status, insurance type, and race. Body mass index (BMI) was calculated as weight divided by height squared (kg/m^2^). Severity‐of‐illness scores included the Sequential Organ Failure Assessment (SOFA), Acute Physiology Score III (APS III), Oxford Acute Severity of Illness Score (OASIS), Glasgow Coma Scale (GCS), and Charlson Comorbidity Index (CCI). Laboratory variables included hemoglobin (Hb), platelet count (PLT), red blood cell count (RBC), white blood cell count (WBC), albumin (Alb), serum creatinine (Scr), blood urea nitrogen (BUN), triglycerides (TG), total cholesterol (TC), high‐density lipoprotein cholesterol (HDL‐C), alanine aminotransferase (ALT), aspartate aminotransferase (AST), Glu, activated partial thromboplastin time (APTT), and international normalized ratio (INR). Vital signs included systolic blood pressure (SBP), diastolic blood pressure (DBP), and heart rate (HR). The TyG index was calculated using the following formula: ln [fasting TG (mg/dL) × fasting Glu (mg/dL)/2]. Comorbidities included hypertension, hyperlipidemia, diabetes, heart failure, myocardial infarction, ischemic heart disease, and atrial fibrillation. Therapeutic interventions included thrombectomy and thrombolysis. TG and Glu values were extracted from measurements obtained within the first 24 h of the initial ICU admission to minimize potential confounding from in‐hospital treatments.

### 2.4 Clinical Outcome

The primary clinical outcome of this study was ICU readmission during hospitalization.

### 2.5 Statistical Analysis

Missing laboratory values were imputed using the k‐nearest neighbor method. This approach was selected to retain the maximum number of eligible patients while minimizing the bias inherent in complete‐case analysis within retrospective critical care databases. Continuous variables were presented as the mean ± standard deviation [SD] or median (interquartile range [IQR]), according to data distribution, whereas categorical variables were presented as frequencies (percentages). Group comparisons were performed using the Student's *t*‐test, Mann–Whitney *U* test, chi‐square test, or Fisher's exact test, as appropriate. Least Absolute Shrinkage and Selection Operator (LASSO) regression with 10‐fold cross‐validation was used for feature selection. Multivariable logistic regression models were constructed to assess the association between the TyG index, analyzed as both quartiles and and a continuous variable, and ICU readmission, adjusting for variables through LASSO regression, including age, sex, BMI, heart failure, ischemic heart disease, diabetes, HR, Hb, Alb, HDL‐C, SOFA, APS III, OASIS, and thrombectomy. Variables with a variance inflation factor (VIF) of > 5 were excluded to address multicollinearity. The receiver operating characteristic curve (AUC) was calculated to evaluate the predictive performance of the TyG index. Decision curve analysis (DCA) was performed, and the net reclassification improvement (NRI) and integrated discrimination improvement (IDI) were calculated to determine whether incorporating the TyG index improved the predictive performance and clinical utility of existing scoring tools and reference models. Restricted cubic spline (RCS) analysis was used to assess nonlinear associations between the TyG index and clinical outcomes. Threshold effect analysis was conducted to identify inflection points. Subgroup analyses were performed, and interaction tests were conducted to assess effect modification. Statistical significance was defined as a two‐sided *p* < 0.05. All analyses were performed using DecisionLinnc 1.0 [14], SAS version 9.4, and R version 4.2.2.

## 3 Results

The mean age of the enrolled patients was 68.5 ± 15.9 years. The cohort included 1471 (50.2%) male and 1458 (49.8%) female patients. The mean TyG index for the overall population was 9.0 ± 0.7. The in‐hospital ICU readmission rate was 24.0%.

### 3.1 Baseline Characteristics

The baseline characteristics of critically ill patients with IS stratified by TyG index quartiles are presented in Table 1. Patients were categorized into quartiles according to TyG index values measured on the first day of ICU admission (Q1: 7.1–8.6; Q2: 8.6–9.0; Q3: 9.0–9.4; Q4: 9.4–13.1). Patients in higher TyG index quartiles generally had significantly higher BMI and severity‐of‐illness scores; a higher prevalence of diabetes, myocardial infarction, and ischemic heart disease; and progressively lower age and prevalence of atrial fibrillation. Laboratory and vital sign values demonstrated significant trends across increasing TyG index quartiles, including increases in WBC, Scr, BUN, TG, TC, ALT, AST, and HR, as well as decreases in Alb and HDL‐C. Patients in higher TyG index quartiles showed progressively increased ICU readmission rates (Q1, 16.3%; Q2, 23.8%; Q3, 26.6%; Q4, 29.5%; *p* < 0.001).

**TABLE 1:** Baseline characteristics and outcomes of patients grouped according to TyG index quartiles.^a^

Categories	Overall (*N* = 2929)	Q1 (*N* = 732)	Q2 (*N* = 732)	Q3 (*N* = 732)	Q4 (*N* = 733)	*p*
Age, years	68.5 (15.9)	70.5 (16.5)	69.5 (15.8)	68.2 (16.2)	65.6 (14.4)	< 0.001
Male, *n* (%)	1471 (50.2)	352 (48.1)	365 (49.9)	372 (50.8)	382 (52.1)	0.47
BMI, kg/m^2^	28.3 (7.8)	26.7 (6.8)	27.5 (6.6)	29.0 (9.3)	30.1 (7.8)	< 0.001
Marital status, *n* (%)						0.04
Married	1440 (49.2)	365 (49.9)	359 (49.0)	335 (45.8)	381 (52.0)	
Single	825 (28.2)	198 (27.0)	196 (26.8)	214 (29.2)	217 (29.6)	
Divorced	237 (8.1)	52 (7.1)	66 (9.0)	62 (8.5)	57 (7.8)	
Widowed	427 (14.6)	117 (16.0)	111 (15.2)	121 (16.5)	78 (10.6)	
Insurance, *n* (%)						0.006
Medicaid	413 (14.7)	91 (12.7)	110 (15.5)	94 (13.6)	118 (17.1)	
Medicare	1595 (56.8)	446 (62.3)	401 (56.5)	403 (58.5)	345 (49.9)	
Private	741 (26.4)	169 (23.6)	183 (25.8)	177 (25.7)	212 (30.6)	
Others	180 (6.1)	26 (3.6)	38 (5.2)	58 (7.9)	58 (7.9)	
Race, *n* (%)						0.20
White	1606 (55.8)	416 (56.8)	416 (57.5)	399 (56.4)	375 (52.4)	
Black	303 (10.5)	81 (11.1)	78 (10.8)	58 (8.2)	86 (12.0)	
Hispanic	100 (3.5)	19 (2.6)	23 (3.2)	31 (4.4)	27 (3.8)	
Asian	90 (3.1)	19 (2.6)	28 (3.9)	21 (3.0)	22 (3.1)	
Others	830 (28.3)	197 (26.9)	187 (25.5)	223 (30.5)	223 (30.4)	
SOFA	3.8 (3.1)	3.1 (2.5)	3.4 (2.8)	3.9 (3.1)	4.8 (3.7)	< 0.001
APSIII	40.9 (19.2)	36.8 (16.2)	39.2 (17.6)	41.1 (19.0)	46.6 (22.0)	< 0.001
OASIS	31.4 (8.4)	29.9 (7.6)	31.0 (8.1)	31.5 (8.7)	33.2 (8.6)	< 0.001
GCS	12.7 (3.0)	12.7 (2.8)	12.7 (3.0)	12.7 (3.1)	12.7 (3.3)	0.02
CCI	6.1 (2.7)	6.1 (2.6)	6.1 (2.7)	6.1 (2.8)	6.2 (2.7)	0.96
Laboratory data						
Hb, g/dL	11.8 (2.3)	11.9 (2.2)	11.9 (2.3)	11.8 (2.3)	11.7 (2.5)	0.12
PLT, K/uL	221.8 (97.6)	220.0 (105.2)	216.2 (77.9)	223.2 (93.2)	227.9 (110.6)	0.11
RBC, m/uL	4.0 (0.8)	4.0 (0.7)	4.0 (0.8)	4.0 (0.8)	4.0 (0.8)	0.46
WBC, K/uL	11.6 (8.7)	9.7 (4.5)	11.1 (9.4)	12.1 (7.7)	13.5 (11.4)	< 0.001
Alb, g/dL	3.4 (0.6)	3.4 (0.6)	3.4 (0.6)	3.4 (0.6)	3.3 (0.7)	< 0.001
Scr, mg/dL	1.2 (1.2)	1.0 (1.0)	1.1 (1.1)	1.2 (1.0)	1.4 (1.6)	< 0.001
BUN, mg/dL	21.3 (16.2)	18.1 (11.7)	19.9 (14.3)	21.9 (17.3)	25.2 (19.7)	< 0.001
TC, mg/dL	161.3 (39.9)	153.4 (39.3)	160.5 (38.2)	162.3 (36.4)	168.9 (43.8)	< 0.001
HDL‐C, mg/dL	46.4 (13.4)	52.1 (15.7)	47.5 (13.3)	44.4 (10.7)	41.7 (10.8)	< 0.001
ALT, U/L	27.3 (17.0)	24.6 (16.3)	27.2 (16.9)	28.1 (16.8)	29.5 (17.9)	< 0.001
AST, U/L	35.4 (21.4)	31.3 (19.1)	36.5 (22.7)	37.1 (21.5)	37.0 (21.8)	< 0.001
Glu, mg/dL	143.7 (65.3)	107.5 (24.3)	120.4 (28.5)	144.5 (42.3)	202.2 (92.6)	< 0.001
TG, mg/dL	144.9 (143.3)	77.5 (22.2)	114.1 (25.2)	139.6 (34.8)	248.4 (252.0)	< 0.001
TyG	9.0 (0.7)	8.3 (0.3)	8.8 (0.1)	9.2 (0.1)	9.9 (0.5)	< 0.001
APTT, s	34.7 (19.3)	34.9 (19.3)	35.2 (19.4)	34.4 (19.4)	34.5 (19.1)	0.11
INR	1.3 (0.4)	1.3 (0.4)	1.3 (0.4)	1.3 (0.3)	1.3 (0.4)	0.72
Vital signs						
SBP, mm Hg	133.4 (20.9)	133.8 (15.3)	133.5 (18.6)	133.5 (22.4)	132.7 (25.9)	0.37
DBP, mm Hg	64.3 (11.2)	64.4 (8.2)	64.4 (10.2)	64.6 (11.5)	64.0 (14.1)	0.31
HR, beats/min	83.7 (19.1)	80.3 (18.7)	81.9 (18.8)	84.8 (18.8)	88.0 (19.0)	< 0.001
Comorbidities, *n* (%)						
Hypertension	1566 (53.5)	379 (51.8)	406 (55.5)	376 (51.4)	405 (55.3)	0.24
Hyperlipidemia	1374 (46.9)	324 (44.3)	367 (50.1)	321 (43.9)	362 (49.4)	0.12
Diabetes	889 (30.9)	109 (14.9)	165 (22.8)	237 (33.5)	378 (52.8)	< 0.001
Heart failure	638 (21.8)	145 (19.8)	159 (21.7)	155 (21.2)	179 (24.4)	0.18
Myocardial infarction	242 (8.3)	30 (4.1)	62 (8.5)	63 (8.6)	87 (11.9)	< 0.001
Ischemic heart disease	869 (29.7)	183 (25.0)	221 (30.2)	219 (29.9)	246 (33.6)	0.004
Atrial fibrillation	709 (24.2)	211 (28.8)	174 (23.8)	169 (23.1)	155 (21.1)	0.005
Treatment						
Thrombectomy, *n* (%)	58 (2.0)	16 (2.2)	19 (2.6)	13 (1.8)	10 (1.4)	0.46
Thrombolysis, *n* (%)	147 (5.0)	41 (5.6)	32 (4.4)	42 (5.7)	32 (4.4)	0.37
Events						
ICU readmission, *n* (%)	704 (24.0)	119 (16.3)	174 (23.8)	195 (26.6)	216 (29.5)	< 0.001
28‐day ICU mortality, *n* (%)	487 (16.6)	75 (10.2)	106 (14.5)	128 (17.5)	178 (24.3)	< 0.001
28‐day in‐hospital mortality, *n* (%)	472 (16.1)	73 (10.0)	103 (14.1)	126 (17.2)	170 (23.2)	< 0.001

*Note:* Values are presented as the mean (± standard deviation [SD]), median (interquartile range [IQR]), or number of participants (%).

Abbreviations: Alb, albumin; Scr, serum creatinine; ALT, alanine aminotransferase; APS III, Acute Physiology Score III; APTT, activated partial thromboplastin time; AST, aspartate aminotransferase; BMI, body mass index; BUN, blood urea nitrogen; CCI, Charlson Comorbidity Index; DBP, diastolic blood pressure; GCS, Glasgow Coma Scale; Glu, glucose; TG, triglycerides; Hb, hemoglobin; HDL‐C, high‐density lipoprotein cholesterol; HR, heart rate; ICU, intensive care unit; INR, international normalized ratio; OASIS, Oxford Acute Severity of Illness Score; PLT, platelet count; RBC, red blood cell count; SBP, systolic blood pressure; SOFA, Sequential Organ Failure Assessment; TC, total cholesterol; TyG, triglyceride glucose index; WBC, white blood cell count.

^a^ TyG index, Q1 (7.1–8.6), Q2 (8.6–9.0), Q3 (9.0–9.4), Q4 (9.4–13.1).

Compared with patients without ICU readmission (*n* = 2225), those with ICU readmission (*n* = 704) were younger, had a higher proportion of males, higher severity‐of‐illness scores, a greater prevalence of comorbidities, and worse laboratory profiles (Table 2; all *p* < 0.05).

**TABLE 2:** Baseline characteristics of patients grouped according to in‐hospital ICU readmission.

Categories	Overall (*N* = 2929)	No (*N* = 2225)	Yes (*N* = 704)	*p*
Age, years	68.5 (15.9)	69.0 (15.8)	66.6 (16.0)	< 0.001
Male, *n* (%)	1471 (50.2)	1094 (49.2)	377 (53.6)	0.04
BMI, kg/m^2^	28.3 (7.8)	28.3 (8.1)	28.4 (6.9)	0.42
Marital status, *n* (%)				0.001
Married	1440 (49.2)	1089 (48.9)	351 (49.9)	
Single	825 (28.2)	606 (27.2)	219 (31.1)	
Divorced	237 (8.1)	175 (7.9)	62 (8.8)	
Widowed	427 (14.6)	355 (16.0)	72 (10.2)	
Insurance, *n* (%)				0.16
Medicaid	413 (14.7)	310 (14.3)	103 (15.9)	
Medicare	1595 (56.8)	1254 (58.0)	341 (52.8)	
Private	741 (26.4)	556 (25.7)	185 (28.6)	
Others	180 (6.1)	105 (4.7)	75 (1.1)	
Race, *n* (%)				0.009
White	1606 (55.8)	1226 (55.1)	380 (58.2)	
Black	303 (10.5)	227 (10.2)	76 (11.6)	
Hispanic	100 (3.5)	75 (3.4)	25 (3.8)	
Asian	90 (3.1)	62 (2.8)	28 (4.3)	
Others	830 (28.3)	635 (25.1)	195 (27.7)	
SOFA	3.8 (3.1)	3.6 (3.0)	4.4 (3.3)	< 0.001
APSIII	40.9 (19.2)	39.9 (19.1)	44.2 (19.1)	< 0.001
OASIS	31.4 (8.4)	31.3 (8.4)	31.8 (8.4)	0.07
GCS	12.7 (3.0)	12.7 (3.0)	12.7 (3.0)	0.71
CCI	6.1 (2.7)	6.1 (2.7)	6.3 (2.7)	0.06
Laboratory data				
Hb, g/dL	11.8 (2.3)	12.0 (2.3)	11.2 (2.3)	< 0.001
PLT, K/uL	221.8 (97.6)	223.0 (89.7)	218.1 (119.2)	0.03
RBC, m/uL	4.0 (0.8)	4.0 (0.8)	3.8 (0.8)	< 0.001
WBC, K/uL	11.6 (8.7)	11.4 (7.7)	12.3 (11.5)	0.005
Alb, g/dL	3.4 (0.6)	3.4 (0.6)	3.2 (0.6)	< 0.001
Scr, mg/dL	1.2 (1.2)	1.1 (1.1)	1.3 (1.5)	0.32
BUN, mg/dL	21.3 (16.2)	20.6 (15.4)	23.3 (18.5)	< 0.001
TC, mg/dL	161.3 (39.9)	162.9 (41.0)	156.3 (35.6)	0.002
HDL‐C, mg/dL	46.4 (13.4)	47.0 (13.6)	44.5 (12.5)	< 0.001
ALT, U/L	27.3 (17.0)	27.1 (17.0)	27.9 (17.2)	0.28
AST, U/L	35.4 (21.4)	34.9 (21.6)	36.9 (20.8)	0.001
Glu, mg/dL	143.7 (65.3)	142.1 (66.0)	148.5 (62.8)	< 0.001
TG, mg/dL	144.9 (143.3)	141.2 (137.4)	156.7 (159.9)	< 0.001
TyG index	9.0 (0.7)	9.0 (0.7)	9.1 (0.6)	< 0.001
APTT, s	34.7 (19.3)	34.7 (19.6)	34.9 (18.2)	0.01
INR	1.3 (0.4)	1.3 (0.4)	1.3 (0.5)	< 0.001
Vital signs				
SBP, mm Hg	133.4 (20.9)	134.1 (20.4)	131.2 (22.5)	0.001
DBP, mm Hg	64.3 (11.2)	64.6 (11.1)	63.7 (11.6)	0.09
HR, beats/min	83.7 (19.1)	82.4 (18.5)	87.9 (20.1)	< 0.001
Comorbidities, *n* (%)				
Hypertension	1566 (53.5)	1222 (54.9)	344 (48.9)	0.005
Hyperlipidemia	1374 (46.9)	1046 (47.0)	328 (46.6)	0.85
Diabetes	889 (30.9)	660 (29.7)	229 (35.1)	0.009
Heart failure	638 (21.8)	429 (19.3)	209 (29.7)	< 0.001
Myocardial infarction	242 (8.3)	165 (7.4)	77 (10.9)	0.003
Ischemic heart disease	869 (29.7)	625 (28.1)	244 (34.7)	< 0.001
Atrial fibrillation	709 (24.2)	529 (23.8)	180 (25.6)	0.33
Treatment				
Thrombectomy, *n* (%)	58 (2.0)	49 (2.2)	9 (1.3)	0.13
Thrombolysis, *n* (%)	147 (5.0)	118 (5.3)	29 (4.1)	0.21

*Note:* Values are mean (± standard deviation [SD]) or number of participants (%).

Abbreviations: Alb, albumin; ALT, alanine aminotransferase; APS III, Acute Physiology Score III; APTT, activated partial thromboplastin time; AST, aspartate aminotransferase; BMI, body mass index; BUN, blood urea nitrogen; CCI, Charlson Comorbidity Index; DBP, diastolic blood pressure; GCS, Glasgow Coma Scale; Glu, glucose; Hb, hemoglobin; HDL‐C, high‐density lipoprotein cholesterol; HR, heart rate; ICU, intensive care unit; INR, international normalized ratio; OASIS, Oxford Acute Severity of Illness Score; PLT, platelet count; RBC, red blood cell count; SBP, systolic blood pressure; Scr, serum creatinine; SOFA, Sequential Organ Failure Assessment; TC, total cholesterol; TG, triglycerides; TyG, triglyceride glucose index; WBC, white blood cell count.

### 3.2 Feature Selection

**FIGURE 2:**
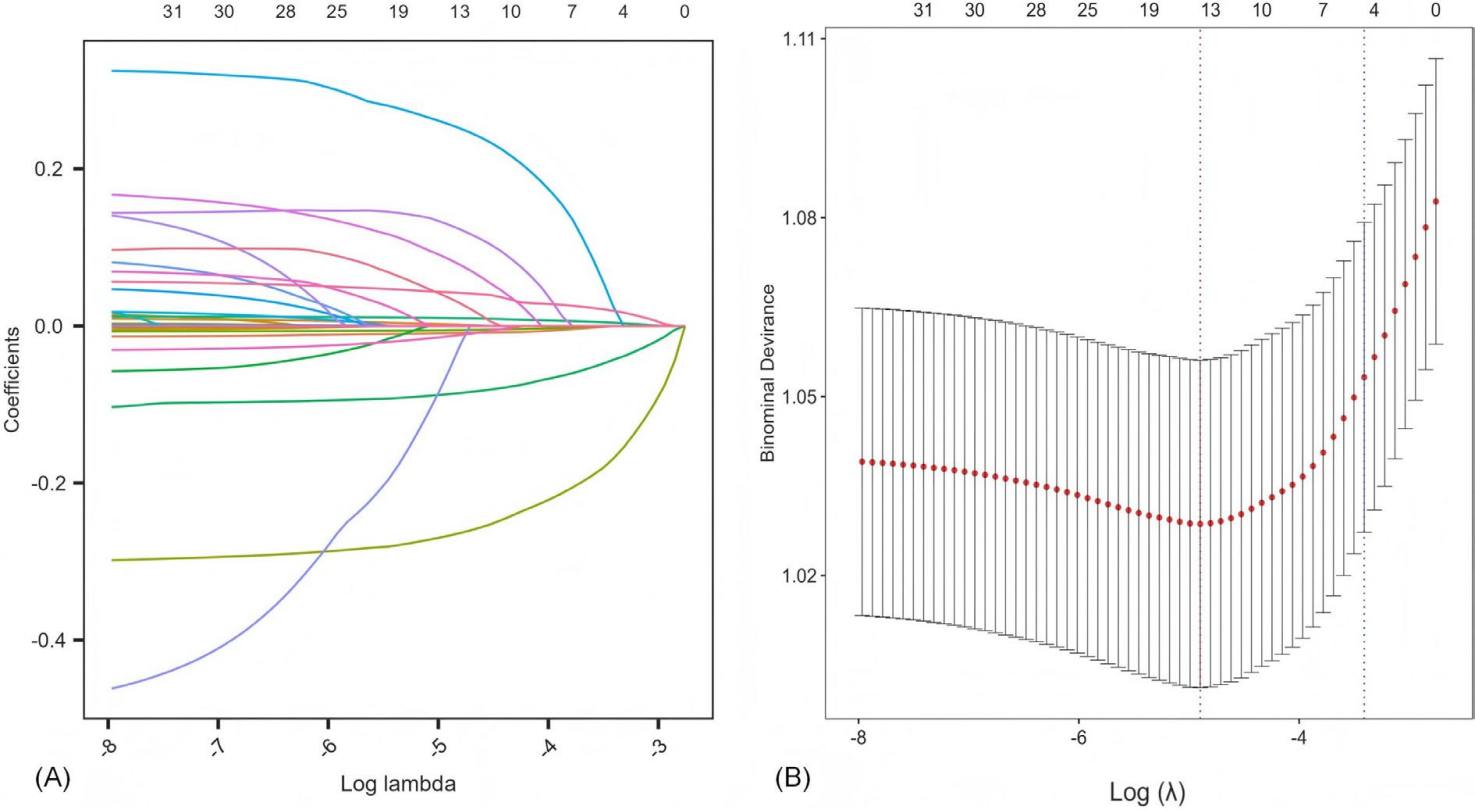
Lasso regression‐based variable screening. (A) Variation characteristics of variable coefficients; (B) The process of selecting the optimal value of the parameter λ in the lasso regression model is carried out by the cross‐validation method.

LASSO regression selected 14 variables from 39 candidate variables for multivariable adjustment: age, sex, BMI, heart failure, ischemic heart disease, diabetes, HR, Hb, Alb, HDL‐C, SOFA, APS III, OASIS, and thrombectomy (Table S1 and Figure 2).

### 3.3 Association Between TyG Index and In‐Hospital ICU Readmission

**FIGURE 3:**
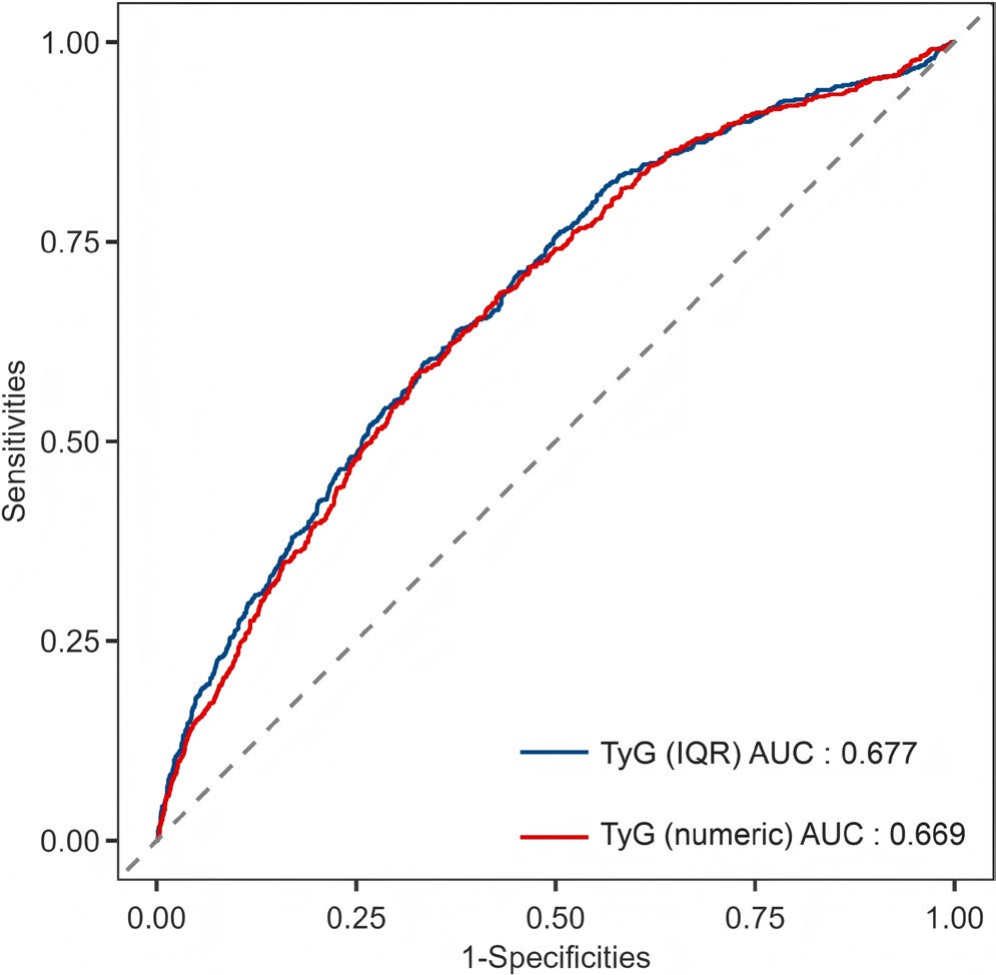
ROC curve analysis of TyG index predicting ICU readmission. ICU, intensive care unit; IQR, interquartile range; ROC, receiver operating characteristic; TyG, triglyceride glucose index.

Logistic regression analysis (Table 3) showed that each 1‐unit increase in the TyG index was associated with a 17% higher risk of ICU readmission in the fully adjusted model (odds ratio [OR], 1.17; 95% confidence interval [CI], 1.01–1.36; *p* = 0.04). Compared with Q1, the risk increased progressively across quartiles (Q2: OR, 1.49; 95% CI, 1.13–1.96; Q3: OR, 1.46; 95% CI, 1.10–1.95; Q4: OR, 1.53; 95% CI, 1.14–2.05; *P* for trend = 0.02). None of the variables in the logistic regression model demonstrated substantial multicollinearity (VIF < 5), and the adjusted GVIF(1/(2 × Df)) < 2 (Table S2). The AUC for the TyG index was 0.677 when analyzed as a continuous variable and 0.669 when analyzed as a categorical variable (Figure 3).

**TABLE 3:** Logistic regression analysis of the association between the TyG index and in‐hospital ICU readmission.

Factor	Model 1			Model 2			Model 3		
	OR (95% CI)	*p*	*p* for trend	OR (95% CI)	*p*	*p* for trend	OR (95% CI)	*p*	*p* for trend
TyG									
Continuous variable per 1 unit	1.41 (1.24–1.59)	< 0.001		1.35 (1.18–1.54)	< 0.001		1.17 (1.01–1.36)	0.04	
Quartile^a^			< 0.0001			< 0.0001			0.02
Q1 (*N* = 732)	Reference			Reference			Reference		
Q2 (*N* = 732)	1.61 (1.24–2.09)	0.0004		1.52 (1.17–1.98)	0.002		1.49 (1.13–1.96)	0.005	
Q3 (*N* = 732)	1.87 (1.45–2.42)	< 0.0001		1.63 (1.25–2.12)	0.0003		1.46 (1.10–1.95)	0.008	
Q4 (*N* = 733)	2.15 (1.67–2.78)	< 0.0001		1.95 (1.51–2.53)	< 0.0001		1.53 (1.14–2.05)	0.005	

*Note:* Model 1: unadjusted. Model 2: adjusted for age, sex, BMI. Model 3: adjusted for age, sex, BMI, heart failure, ischemic heart disease, heart rate, diabetes, hemoglobin, albumin, HDL‐C, SOFA, APS III, OASIS, and thrombectomy.

Abbreviations: OR, odds ratio; TyG, triglyceride glucose index.

^a^ TyG index, Q1 (7.1–8.6), Q2 (8.6–9.0), Q3 (9.0–9.4), Q4 (9.4–13.1).

**FIGURE 4:**
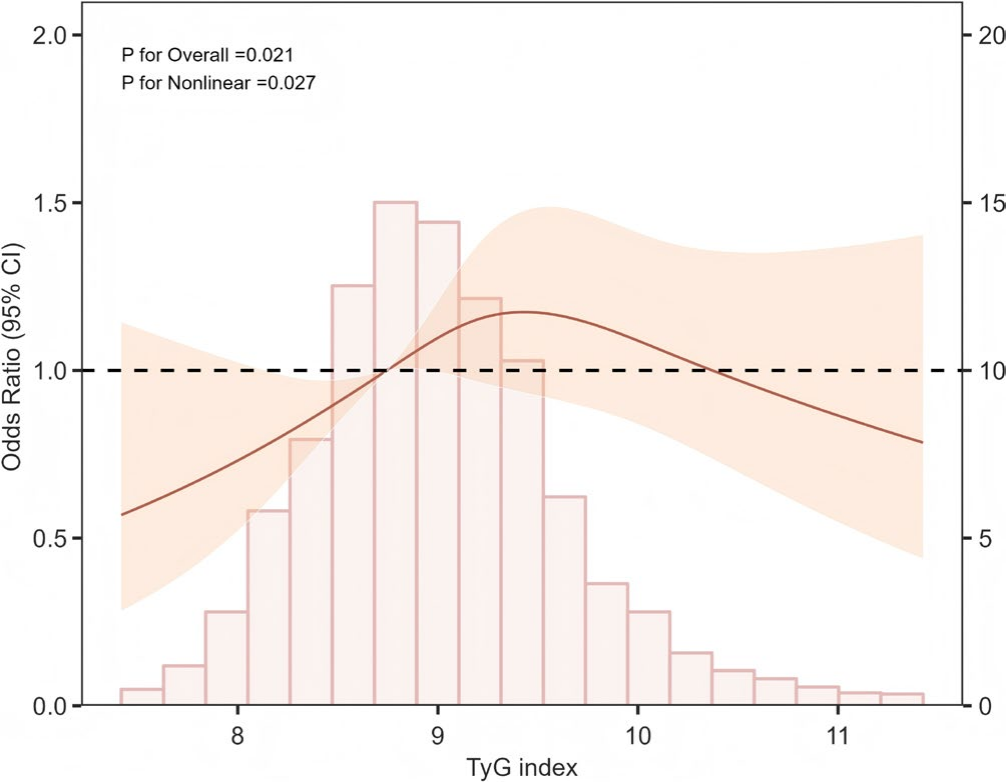
RCS curves for TyG index and odds ratio. Heavy central lines represent the estimated adjusted odds ratios, with shaded ribbons denoting 95% confidence intervals. The horizontal dashed lines represent the odds ratio of 1.0. CI, confidence interval; OR, odds ratio; RCS, restricted cubic spline; TyG, triglyceride glucose index.

RCS analysis (Figure 4) revealed a significant nonlinear association between the TyG index and the risk of ICU readmission (*P* for nonlinearity = 0.027). Threshold effect analysis (Table S3) identified an inflection point at a TyG index value of 9.82 (*P* for the log‐likelihood ratio = 0.007). When the TyG index was < 9.82, each 1‐unit increase in the TyG index was associated with a 33% higher risk of ICU readmission (OR, 1.33; 95% CI, 1.09–1.63; *p* = 0.006).

### 3.4 Incremental Effect of the TyG Index for Predicting ICU Readmission

**FIGURE 5:**
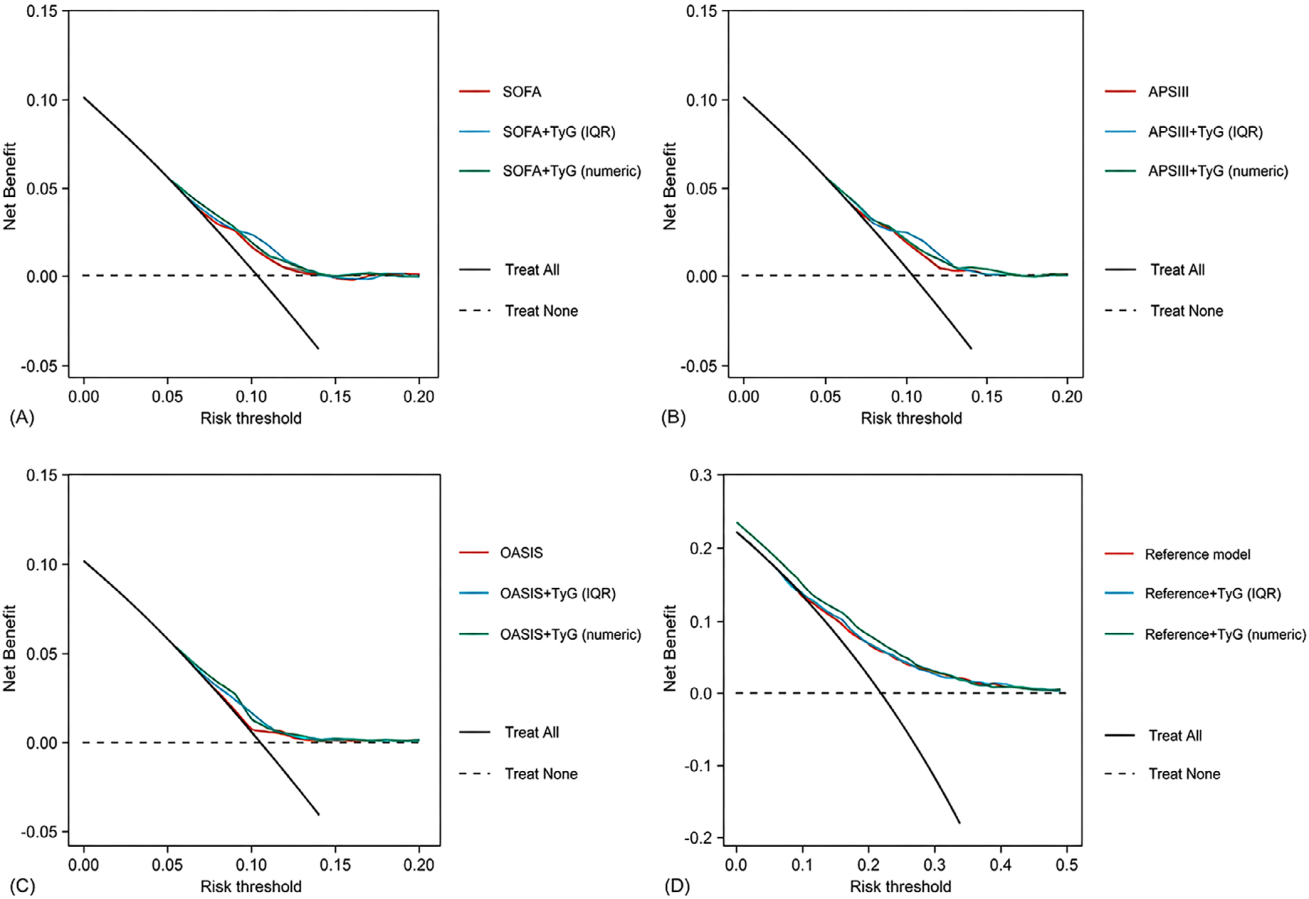
DCA of scoring tools and predicting model with and without considering the TyG index.

In addition, we calculated the NRI and IDI of severity scoring tools (APS III, OASIS, and SAPS II) and the reference model after incorporating the TyG index. The reference model included age, sex, BMI, heart failure, ischemic heart disease, diabetes, HR, Hb, Alb, HDL‐C, SOFA, APS III, OASIS, and thrombectomy. The integration of the TyG index, analyzed as either a categorical variable (TyG‐IQR) or a continuous variable (TyG‐numeric), into the severity scores (SOFA, APS III, and OASIS) and the reference model improved predictive performance for in‐hospital ICU readmission, as reflected by changes in AUC, NRI, and IDI (Table 4). For the severity scores (SOFA, APS III, and OASIS), TyG‐IQR increased the AUC by 0.028 to 0.043 and significantly improved reclassification, as demonstrated by significant NRI and IDI values (*p* ≤ 0.0003). Continuous TyG improved the AUC by 0.013 to 0.048, with NRI and IDI reaching statistical significance (*p* ≤ 0.02) across all severity scores except APS III, which the NRI did not reach statistical significance (0.097, *p* = 0.11). For the reference model, TyG‐IQR increased the AUC and significantly improved both the NRI and IDI (*p* ≤ 0.001). DCA demonstrated an enhanced net clinical benefit for the severity scores (SOFA, APS III, and OASIS) and the reference model after incorporation of the TyG index (Figure 5).

**TABLE 4:** Incremental effect of TyG for predicting ICU readmission.

	AUC	NRI (95% CI)	*p*	IDI (95% CI)	*p*
Score					
SOFA	0.580	Reference		Reference	
+TyG (IQR)	0.608	0.185 (0.112, 0.259)	< 0.0001	0.004 (0.006, 0.013)	< 0.0001
+TyG (numeric)	0.603	0.130 (0.045, 0.214)	0.003	0.005 (0.002, 0.008)	0.0003
APS III	0.604	Reference		Reference	
+TyG (IQR)	0.618	0.194 (0.102, 0.285)	< 0.0001	0.004 (0.002, 0.006)	0.0003
+TyG (numeric)	0.617	0.097 (−0.022, 0.215)	0.11	0.004 (0.001, 0.007)	0.004
OASIS	0.528	Reference		Reference	
+TyG (IQR)	0.571	0.211 (0.120, 0.303)	< 0.0001	0.005 (0.003, 0.008)	< 0.0001
+TyG (numeric)	0.576	0.140 (0.021, 0.259)	0.02	0.006 (0.003, 0.010)	0.0003
Reference model	0.670	Reference		Reference	
+TyG (IQR)	0.677	0.200 (0.127, 0.274)	< 0.0001	0.005 (0.003, 0.008)	0.0001
+TyG (numeric)	0.669	0.093 (0.008, 0.177)	0.03	0.001 (−0.001, 0.003)	0.11

*Note:* Reference model included age, sex, BMI, heart failure, ischemic heart disease, heart rate, diabetes, hemoglobin, albumin, HDL‐C, SOFA, APS III, OASIS, and thrombectomy.

Abbreviations: APSIII, Acute Physiology Score III; AUC, area under the curve; CI, confidence interval; IDI, integrated discrimination improvement; IQR, interquartile range; NRI, net reclassification improvement; OASIS, Oxford Acute Severity of Illness Score; SOFA, Sequential Organ Failure Assessment; TyG, triglyceride glucose index.

### 3.5 Subgroup Analysis

**FIGURE 6:**
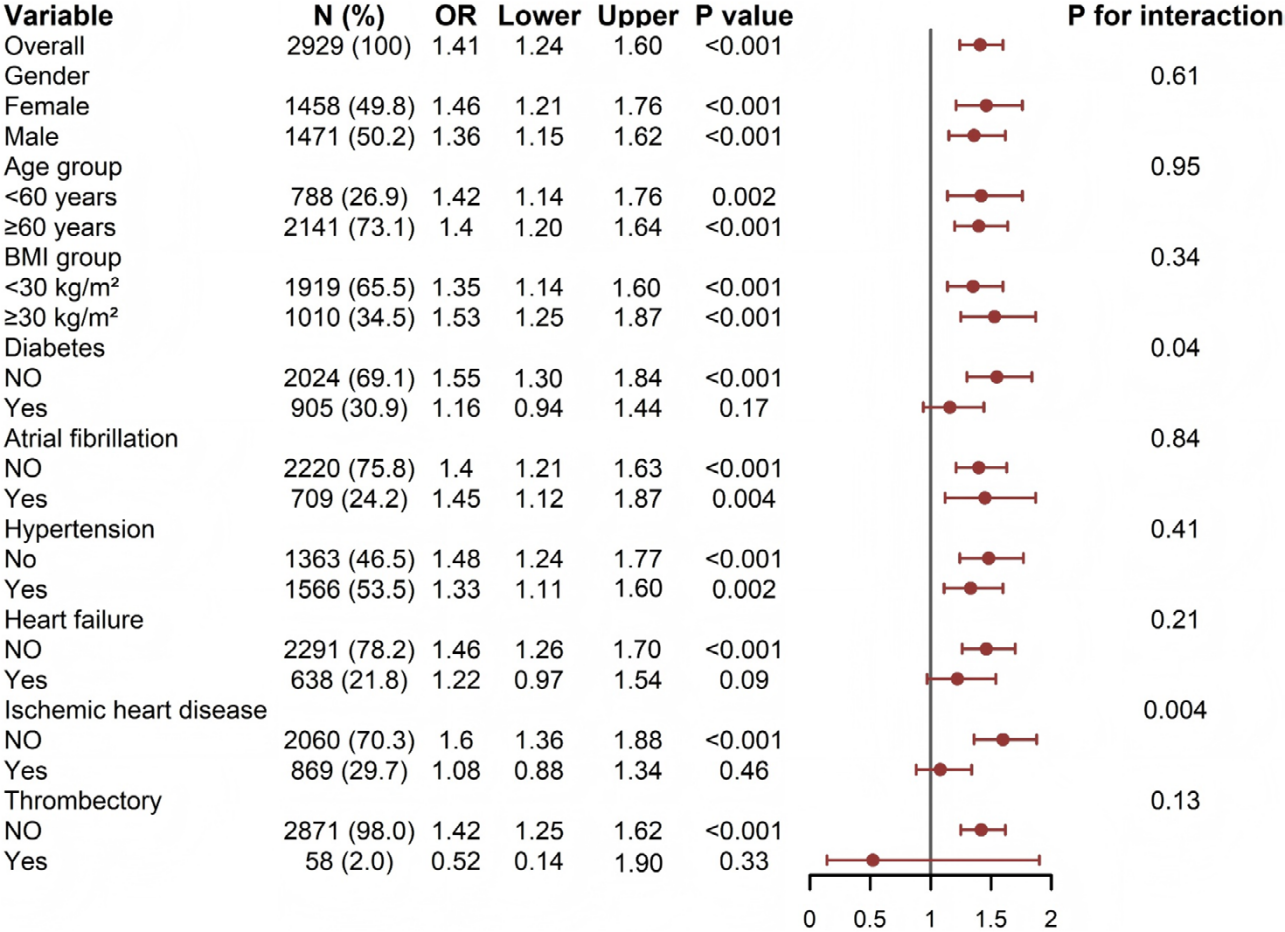
Subgroup analyses for the association of TyG index with ICU readmission. OR, odds ratio.

Subgroup analyses demonstrated that an elevated TyG index was significantly associated with an increased risk of ICU readmission across multiple clinical subgroups, including age (< 60 and ≥ 60 years), sex (female and male), BMI (< 30 and ≥ 30 kg/m^2^), atrial fibrillation status, and hypertension status (*p* ≤ 0.002; Figure 6). The association remained statistically significant among patients without diabetes, heart failure, ischemic heart disease, or thrombectomy (all *p* < 0.001) but was not statistically significant among patients with these conditions (all *p* > 0.05). Most subgroup comparisons showed no significant effect modification (all *p* for interaction > 0.05), except for ischemic heart disease (*p* for interaction = 0.004) and diabetes (*p* for interaction = 0.04).

## 4 Discussion

This study demonstrated that the TyG index was associated with in‐hospital ICU readmission in patients with IS. After adjustment, each 1‐unit increase in the TyG index was associated with a 17% higher risk, and patients in the highest TyG index quartile (≥ 9.4) had a 53% higher risk of ICU readmission compared with those in the lowest quartile. We also observed a significant nonlinear association, with an inflection point at a TyG index value of 9.82. Incorporating the TyG index into established severity scores (SOFA, APS III, and OASIS) and the reference model improved predictive performance.

IR contributes to cardiovascular disease [15]. Although the homeostasis model assessment of insulin resistance (HOMA‐IR) is commonly used to estimate IR, the TyG index provides a more efficient, cost‐effective, and reproducible alternative [16]. An elevated TyG index has been associated with cardiovascular and cerebrovascular diseases and may outperform HOMA‐IR in reflecting IR [17, 18, 19, 20].

Substantial evidence has demonstrated that the TyG index is associated with both the occurrence and prognosis of IS, including stroke recurrence and early neurological deterioration [6, 8, 21, 22]. These findings suggest that the TyG index reflects metabolic dysregulation relevant to disease progression. Critically ill patients with IS are susceptible to acute deterioration and often require recurrent ICU care. ICU readmission is therefore a marker of clinical instability and poor prognosis [11, 12]. In this context, ICU readmission represents a composite indicator of overall physiological vulnerability rather than a single disease‐specific event. Accordingly, the observed association suggests that early metabolic disturbances may contribute to a broad spectrum of post‐stroke complications. Early identification of high‐risk patients based on metabolic status may facilitate timely monitoring and intervention to improve clinical outcomes [23].

Our study found that patients with a higher TyG index had significantly worse laboratory profiles and higher severity scores. Importantly, an elevated TyG index was independently associated with a 17% higher risk of in‐hospital ICU readmission per unit increase and up to a 53% higher risk in the highest quartile. The significant nonlinear association identified by RCS analysis revealed an inflection point at a TyG index value of 9.82. Below this threshold, each 1‐unit increase in the TyG index was associated with a 33% higher risk of readmission, whereas above this value, the association attenuated and was no longer clearly positive. This attenuation may reflect intensified clinical management or the predominance of stress‐induced hyperglycemia at extreme TyG levels. Clinically, these findings suggest that the risk increases most steeply when the TyG index is below 9.82, rather than remaining progressively elevated across the entire exposure range.

Several mechanisms may explain the association between the TyG index and cerebrovascular disease. Patients with acute IS often develop hyperglycemia and IR owing to the stress response. IR may exacerbate stroke progression through interconnected thrombotic and inflammatory pathways [15]. First, IR induces endothelial dysfunction and platelet hyperactivation, thereby compromising cerebral blood flow regulation and accelerating atherosclerotic thrombosis [24, 25]. Second, IR amplifies oxidative stress and chronic inflammation through upregulation of inflammatory genes and mitochondrial dysfunction, thereby damaging blood vessels and contributing to ischemia–reperfusion injury [26]. Third, IR accelerates atherosclerosis by inducing endoplasmic reticulum stress, macrophage apoptosis, and vulnerable plaque formation, thereby increasing the risk of secondary cerebrovascular events [25]. These mechanisms collectively amplify cerebral damage, increase thrombotic risk, and worsen clinical outcomes after IS.

Our analysis also demonstrated that incorporating the TyG index into established ICU severity scores (SOFA, APS III, and OASIS) and a reference model significantly improved the prediction of in‐hospital ICU readmission. These findings are consistent with prior research demonstrating improved risk reclassification when the TyG index was added to conventional predictive models. A large‐scale multicenter study including 3216 patients with acute IS reported that adding the TyG index to a conventional model improved the NRI by 10.37% and the IDI by 0.27% (both *p* < 0.05) [10]. Another retrospective study based on the MIMIC‐IV database, which enrolled 1810 patients undergoing cardiac surgery, showed that a TyG index–based model outperformed established scoring systems in predicting mortality, with significantly positive IDI values for SOFA, APS III, and OASIS [27].

However, the clinical utility of these incremental improvements should be interpreted with caution. The standalone AUC of the TyG index remains within the poor‐to‐fair range; the gain in discrimination beyond the reference model is minimal; and DCA indicates only modest separation between curves. Therefore, the TyG index should be considered a low‐cost and readily available metabolic marker that provides modest incremental prognostic information rather than a replacement for established severity scores.

Subgroup analyses confirmed a consistent association between an elevated TyG index and an increased risk of ICU readmission across subgroups stratified by age, sex, BMI, atrial fibrillation status, and hypertension status. However, this association was not statistically significant among patients with diabetes, heart failure, ischemic heart disease, or those undergoing thrombectomy. The null finding in the diabetic subgroup may reflect more intensive Glu management in these patients, which could mitigate adverse metabolic effects, as well as the difficulty of distinguishing acute metabolic stress from chronic IR [28]. In contrast, among non‐diabetic critically ill patients, stress‐induced hyperglycemia is often more pronounced, and the TyG index in this group may predominantly capture acute metabolic decompensation. Clinicians should integrate the TyG index with clinical features to refine risk stratification during hospitalization.

This study has several limitations. First, this retrospective single‐center design cannot definitively establish causality. Although we adjusted for multiple confounders, residual or unmeasured factors may still have influenced the clinical outcomes. Second, the MIMIC‐IV database does not distinguish between planned and unplanned ICU readmissions or provide specific indications for readmission, which limits mechanistic interpretation of the observed association. Third, only the baseline TyG index was analyzed, and dynamic changes in the TyG index were not evaluated. Therefore, future studies should assess the predictive value of longitudinal changes in the TyG index. Fourth, true fasting status could not be reliably ascertained in this critically ill population, which may have introduced measurement error into the calculation of the TyG index.

## 5 Conclusions

In conclusion, this study demonstrated a significant nonlinear dose–response association between the TyG index and the risk of in‐hospital ICU readmission among patients with IS. Importantly, integrating the TyG index with established prognostic models improved risk stratification accuracy, underscoring its potential clinical utility for the early identification of high‐risk individuals.

## Funding

This work was supported by the Shanghai Municipal Health Commission (20244Y0064).

## Ethics Statement

This study was conducted in accordance with the Declaration of Helsinki. The MIMIC‐IV project was approved by the institutional review boards of the Massachusetts Institute of Technology and Beth Israel Deaconess Medical Center. Patient information was anonymized; therefore, the requirement for informed consent was waived.

## Conflicts of Interest

The authors declare no conflicts of interest.

## Supporting information


**Table S1:** The results of the LASSO regression analysis.
**Table S2:** The results of the collinearity screening analysis.
**Table S3:** Threshold effect analysis of TyG index on ICU readmission.

## Data Availability

The MIMIC‐IV database is publicly available and can be accessed from the website https://mimic.physionet.org/.
