# Supplementary material for: Association Between Triglyceride‐Glucose Index and in‐Hospital Intensive Care Unit Readmission in Ischemic Stroke Patients: A Retrospective Analysis Based on the MIMIC‐IV Database
Source: Kaohsiung J Med Sci. 2026 Jun 16:e70250. Online ahead of print. doi: 10.1002/kjm2.70250 (PMC13399746; doi:10.1002/kjm2.70250)
Supplement: Supplementary file 1 — Table S1: The results of the LASSO regression analysis. Table S2: The results of the collinearity screening analysis. Table S3: Threshold effect analysis of TyG index on ICU readmission. [file KJM2-9999-e70250-s001.docx]

**Table S1.** The results of the LASSO regression analysis

| Variables | Coefficients |
| --- | --- |
| Age | -0.008561792 |
| Gender | 0.096252811143131 |
| BMI | 0.005142865247 |
| Marital status | 0 |
| Insurance | 0 |
| Race | 0 |
| Atrial fibrillation | 0 |
| Hypertension | 0 |
| Hyperlipidemia | 0 |
| Heart failure | 0.256512603098319 |
| Myocardial infarction | 0 |
| Ischemic heart disease | 0.128372269598218 |
| Diabetes | 0.0354402910592897 |
| HR | 0.0100358779815647 |
| SBP | 0 |
| DBP | 0 |
| Hb | -0.086742274 |
| PLT | 0 |
| RBC | 0 |
| WBC | 0 |
| Alb | -0.266827636 |
| Scr | 0 |
| INR | 0 |
| APTT | 0 |
| HDL-C | -0.005436553 |
| TC | 0 |
| ALT | 0 |
| AST | 0 |
| BUN | 0 |
| Thrombolysis | 0 |
| Thrombectomy | -0.055355084 |
| SOFA | 0.0427891194114853 |
| APSIII | 0.00193700524334988 |
| OASIS | -0.013139877 |
| GCS | 0 |
| CCI | 0 |

**Table S2.** The results of the collinearity screening analysis

| VariableName | GVIF | Df | GVIF^(1/(2*Df)) |
| --- | --- | --- | --- |
| Age | 1.28397423330868 | 1 | 1.13312586825501 |
| Gender | 1.15821067701833 | 1 | 1.076201969 |
| BMI | 1.13449068758541 | 1 | 1.06512472865173 |
| Heart failure | 1.18058363103359 | 1 | 1.08654665386885 |
| Ischemic heart disease | 1.18659018483345 | 1 | 1.08930720406754 |
| Diabetes | 1.17493511269671 | 1 | 1.08394423873957 |
| HR | 1.16150120927046 | 1 | 1.07772965500188 |
| Alb | 1.20302748249376 | 1 | 1.09682609491831 |
| HDL-C | 1.14520350320044 | 1 | 1.07014181452761 |
| TyG index | 1.24471229638591 | 3 | 1.03715778126635 |
| Hb | 1.26283769216605 | 1 | 1.12376051370657 |
| Thrombectomy | 1.01120564614078 | 1 | 1.00558721458697 |
| SOFA | 2.51413207245771 | 1 | 1.58560148601649 |
| APSIII | 2.82858628125963 | 1 | 1.68184014735635 |
| OASIS | 2.17711093496759 | 1 | 1.47550362079108 |

GVIF, generalized variance inflation factor; Df, degrees of freedom.

**Table S3.** Threshold effect analysis of TyG index on ICU readmission

|  |  | OR (95% CI) | *P* value |
| --- | --- | --- | --- |
| Standard Linear Regression Model |  | 1.17 (1.01-1.36) | 0.04 |
| Two-stage regression models  inflection point | 9.82 |  |  |
| TyG < 9.82 |  | 1.33 (1.09, 1.63) | 0.006 |
| TyG > 9.82 |  | 0.65 (0.42, 0.99) | 0.05 |
| *P* for log-likelihood ratio |  |  | 0.007 |
